# Nitrous Oxide Gas in Surgery

**Published:** 1870-09

**Authors:** 


### Nitrous Oxide Gas in Surgery.

Dr. Chas. J. Fox, in the London *Lancet*, for July, advocates the use of nitrous oxide gas in general surgery, for the following reasons:—

1. Its safety.

2. The rapidity with which anæsthesia, can be induced, viz., from 50 to 100 seconds.

3. The readiness with which a patient can either be kept for a prolonged period in the anæsthetic state, or, if the surgeon so wills, can be promptly and thoroughly awakened.

4. Because it is actually pleasant to the patient to inhale, and, therefore, much fright and mental distress is avoided, diminishing the danger of death by syncope.

5. Because recovery is usually bright, pleasant and complete, any after discomfort being extremely rare.

6. Because sickness has never, to my knowledge, occurred during the administration of this anæsthetic, and but *rarely* afterwards.

He uses the gas in the form of a solution, known as "Coxeter's Liquid Gas."—*Michigan University Medical Journal.*

